# Photoexcitation dynamics and energy engineering in supramolecular doping of organic conjugated molecules

**DOI:** 10.1038/s41377-022-01062-6

**Published:** 2023-01-31

**Authors:** Xiang An, Chuanxin Wei, Lubing Bai, Jun Zhou, Le Wang, Yamin Han, Lili Sun, Jinyi Lin, Heyuan Liu, Jiewei Li, Man Xu, Haifeng Ling, Linghai Xie, Wei Huang

**Affiliations:** 1 Key Laboratory of Flexible Electronics (KLOFE) & Institute of Advanced Materials (IAM), Nanjing Tech University (NanjingTech), 30 South Puzhu Road, Nanjing, 211816 China; 2 State Key Laboratory of Organic Electronics and Information Displays & Institute of Advanced Materials (IAM), Nanjing University of Posts & Telecommunications, 9 Wenyuan Road, Nanjing, 210023 China; 3 College of Science and Institute of New Energy, China University of Petroleum (East China), Qingdao, 266580 China; 4 Shaanxi Institute of Flexible Electronics (SIFE), Northwestern Polytechnical University (NPU), 127 West Youyi Road, Xi’an, 710072 Shaanxi China

**Keywords:** Optical materials and structures, Lasers, LEDs and light sources

## Abstract

Doping and blending strategies are crucial means to precisely control the excited states and energy level in conjugated molecular systems. However, effective models and platforms are rarely proposed to systematically explore the effects of the formation of trapped doped centers on heterogeneous structures, energy level and ultrafast photophysical process. Herein, for deeply understanding the impact of molecular doping in film energy levels and photoexcitation dynamics, we set a supramolecular N-B coordination composed by the conjugated molecules of pyridine functionalized diarylfluorene (host material), named as ODPF-Phpy and ODPF-(Phpy)_2_, and the molecule of tris(perfluorophenyl)borane (BCF) (guest material). The generation of the molecular-level coordination bond increased the binding energy of N atoms and tuned the band-gap, leading to a new fluorescent emission center with longer excitation wavelength and emission wavelength. The intermolecular Förster resonance energy transfer (FRET) in blending films make it present inconsistent fluorescent behaviors compared to that in solution. The charge transfer (CT) state of N-B coordinated compounds and the changed dielectric constant of blending films resulted in a large PL spectra red-shift with the increased dopant ratio, causing a wide-tunable fluorescent color. The excited state behaviors of two compounds in blending system was further investigated by the transient absorption (TA) spectroscopy. Finally, we found supramolecular coordination blending can effectively improve the films’ photoluminescence quantum yield (PLQY) and conductivity. We believe this exploration in the internal coordination mechanisms would deepen the insights about doped semiconductors and is helpful in developing novel high-efficient fluorescent systems.

## Abstract (web summary)

The supramolecular coordination doping/blending allow for intermolecular Förster resonance energy transfer (FRET) and intramolecular charge transfer (ICT) in doping/blending films, endowing films with tunable bandgap, high PLQY and high conductivity.

## Graphical abstract

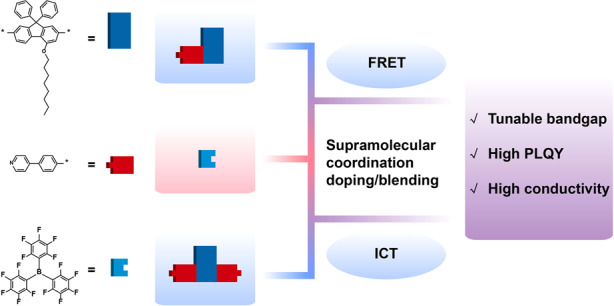

## Introduction

Controlling the energy level and bandgap of organic conjugated molecules is crucial for constructing high-efficient and stable optoelectronic devices, and play a fundamental role in manipulating the charge transport, film conductivity, charge density, and the excited photophysics dynamics^1–9^. Beyond the tailorable chemical structure, physical doping and blending is the convenient and versatile strategy to precisely tune and control the photophysical processing and electrical properties of conjugated molecular systems^10–15^. Additionally, the dopants system generally exhibited exceptional/novel optoelectronic properties that the parent active conjugated molecules don’t possess^15–18^. For optoelectronic materials, the effects of dopants mainly concerned the energy transfer, electron transfer and carrier transport, which predictably control the fundamental photophysical properties and behaviors, and further improve the macroscopic emission efficiency, conductivity and charge carrier mobility/density^13–15,17–22^. However, the poor compatibility between guest and host molecules, such as the serious phase separation, tends to largely reduce doped efficiency^13,15,23^. Therefore, efficient dispersion of the dopant in organic “parent matrix” at molecular-level is a key factor to dominate the intensity of electron coupling, charge-transfer, energy hybrid and electron coupling either in the *p*- or *n*- doping of organic semiconductors. Up to date, the weak interaction between the dopant and organic molecules mostly rely on the weak dielectric and electrostatic interaction, even purify physical blend without any recognition and orientation^11,15^. Physical doping based on the conventional method suffers from these problems including reproducibility, bulk homogeneity, and stability, leading substantial obstacles to commercial implementation. More importantly, it is reasonably foreseen that the amplification of doping effect is easily obtained and ensure the effectiveness of dopant tool in heterogeneous conjugated solid states. Besides, although *p*-type dopants showed great promise in solution-processed conjugated materials, the doping mechanisms have not been fully understood, especially the interaction mechanism between dopant and matrix molecules^17,23^. In this work, we focused on the enhancement of dispersion efficiency and reproducibility of doped organic conjugated molecules via dynamic supramolecular approach, and systematically investigated the tunable energy bandgap and levels of doped system via the observation of photo excitation processing.

The π-electron delocalizing along the conjugated backbone of organic semiconductors enable these materials experience complicated intramolecular excitonic or photoelectrical process^24–27^. As a consequence of *p-n* molecular design engineering^28–30^, the unique electronic structure of heteroatoms provides “functional sites” to form non-covalent interactions in single and multi-component systems, and hence facilitates controlling the molecular packing or doping complexes in a heterogeneous condensed structure^18,31^. Thus, the supramolecular dopants that dispersed effectively at molecular level is more efficient in optimizing the energy transfer and charge carrier mobility, avoiding serious phase separation, and amplifying the electron coupling and conductivity^13,32,33^. However, the specific impact resulting from supramolecular “doping” with weak interactions in the excited state, such as polaron pairs, charge transfer states and triplet states, is still unclear. Therefore, exploring the doping and blending mechanisms is significant for understanding the fundamental physical principles of organic optoelectronic materials. However, although a number of interesting phenomenon and applications about the non-metallic coordination blending have been reported previously, the internal mechanism remains to be further studied^18,21,23,34–37^.

**Scheme 1:**
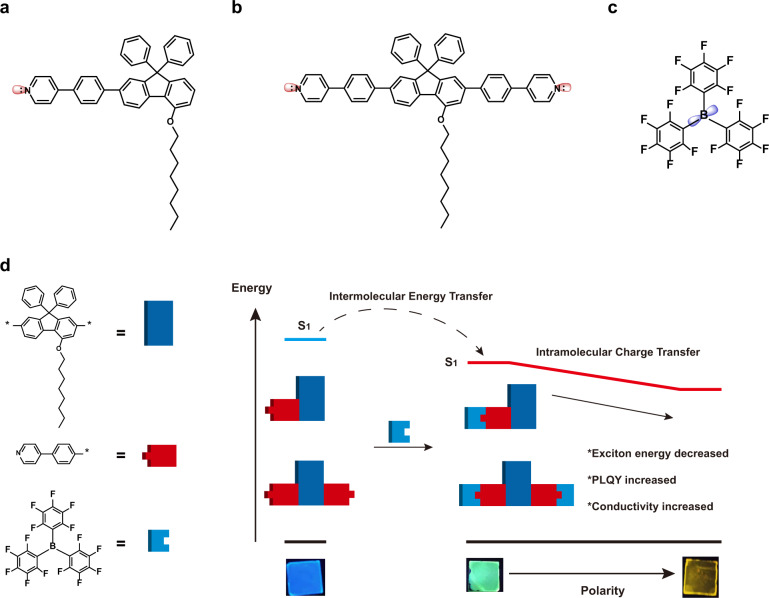
The chemical structures of (**a**) ODPF-Phpy, (**b**) ODPF-(Phpy)_2_ and (**c**) BCF. **d** The excited state behavior in coordination blending films. In blending films, the intermolecular FRET occurred from the original compound to the coordinated compound. For the coordinated compound, the intramolecular charge transfer leads to the exciton energy decreased with film polarity increasing. Insets also present the photographic images of initial spin-coated film and blending films under UV lamp. Emission colors can be easily tuned from deep-blue, to green and orange, also confirmed narrow band-gap and multi-emission species after supramolecular N-B coordination

Here, we investigated the supramolecular N-B coordination doping in organic semiconductor films, obtained the tunable energy level and bandgap via controlling the doped processing. The host light-emitting materials, 4-(4-(5-(octyloxy)-9,9-diphenyl-9H-fluoren-2-yl)phenyl) pyridine (ODPF-Phpy) and 4,4’-((4-(octyloxy)-9,9-diphenyl-9H-fluorene-2,7-diyl)bis(4,1-phenylene))dipyridine (ODPF-(Phpy)_2_) (Figs. S1–S5) were shown in Schemes 1a and 1b, and the “guest” dopant was tris(perfluorophenyl)borane (BCF, Scheme 1c). Firstly, we verified the supramolecular N-B coordination bond in blending films via X-ray photoelectron spectroscopy (XPS) analysis. The two types of excitons of original compound and coordinated compound have quite different optical properties. The intermolecular FRET occurred from the original compound to the coordinated compound in blending films (Scheme 1d). The first-principles density functional theory (DFT) calculations showed that the highest occupied molecular orbital (HOMO) and the lowest unoccupied molecular orbital (LUMO) of coordinated compounds were severely delocalized, leading to the intramolecular charge transfer (ICT) state. The different blending ratio also caused the different polarity in blending films, which was the main reason for the red-shifted PL spectra. We investigated the excited state behavior of the blending films by transient absorption (TA) spectroscopy. Finally, we found the supramolecular coordination blending can effectively improve the photoluminescence quantum yield (PLQY) and conductivity. We believe the exploration of the internal mechanism of coordination blending can provide potential application and development for organic optoelectronics.

## Results

### Supramolecular N-B coordination in blending system

**Fig. 1:**
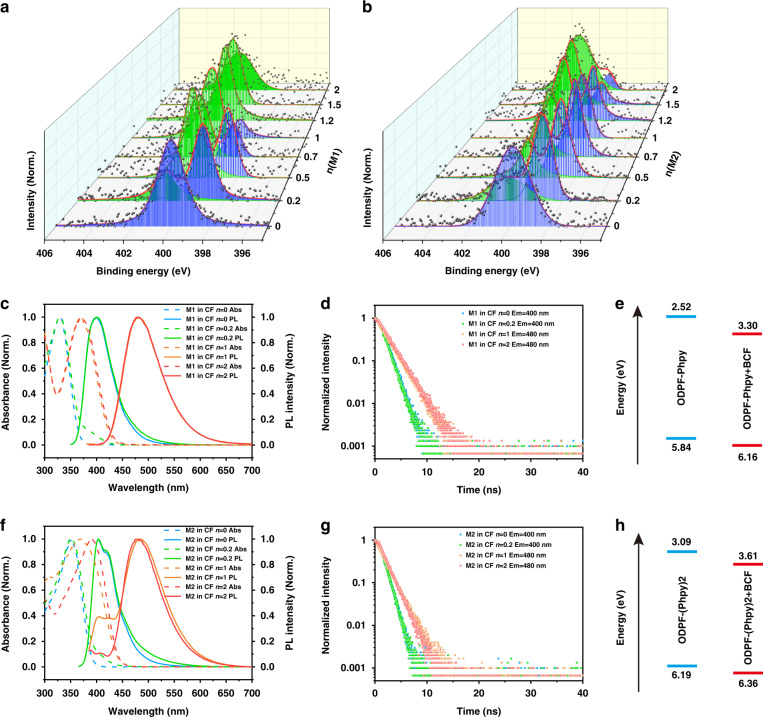
The basic properties of original compounds and coordinated compounds. **a** Binding energy of N-1s region for M1 blending films. **b** Binding energy of N-1s region for M2 blending films. Shades of green: coordinated N atoms. Shades of blue: uncoordinated N atoms. **c** Abs and PL spectra of M1 with *n* = 0, 0.2, 1 and 2 in CF. Concentration: 10^-5 ^mol L^–1^. **d** Fluorescent lifetime of M1 with *n* = 0, 0.2, 1 and 2 in CF (10^-5 ^mol L^–1^). The lifetime was 1.22 ns, 1.24 ns, 2.15 ns and 2.18 ns, respectively. **e** The HOMO and LUMO energy of ODPF-Phpy and ODPF-Phpy + BCF. **f** Abs and PL spectra of M2 with *n* = 0, 0.2, 1 and 2 in CF. Concentration: 10^-5 ^mol L^–1^. **g** Fluorescent lifetime of M2 with *n* = 0, 0.2, 1 and 2 in CF (10^-5 ^mol L^–1^). The lifetime was 1.01 ns, 1.02 ns, 1.79 ns and 1.56 ns, respectively. **h** The HOMO and LUMO energy of ODPF-(Phpy)_2_ and ODPF-(Phpy)_2_ + BCF

The two-component blending systems with different mole ratios were prepared in chloroform (CF). We named ODPF-Phpy blending with BCF as M1 and ODPF-(Phpy)_2_ blending with BCF as M2, and set the mole ratio *n*(BCF):*n*(ODPF-Phpy/ODPF-(Phpy)_2_) as *n*. A distinguishing feature of coordination blending is the generation of coordination bond in the blending system, leading to the change of the binding energy of coordination atoms. In M1 and M2 blending systems, the binding energy of B atoms was not changed obviously but the binding energy of N atoms can be changed by the forming of coordination bond (Figs. S7–S9). As shown in Fig. 1a and 1b, the binding energy intensity of N element relatively enhanced at 402 eV (shades of green) and decreased at 400 eV (shades of blue) with the increase of *n*. The binding energy at 400 eV and 402 eV represents the uncoordinated N atoms and coordinated N atoms, respectively^38^. From the compound structure, the complete N-B coordination occurred when the mole ratio of N to B was 1:1 (M1 with *n* = 1, M2 with *n* = 2). In fact, the complete coordination needs higher *n* that the uncoordinated N atoms still existed in M1 with *n* = 1 and M2 with *n* = 2. As shown in Fig. 1a, the complete coordination occurred when *n* = 1.2 in M1. This result indicated that the blending films need excess BCF to be complete coordination. The binding energy of N atoms showed that the blending films both contain original compound (ODPF-Phpy or ODPF-(Phpy)_2_) and coordinated compound (ODPF-Phpy + BCF or ODPF-(Phpy)_2_ + BCF) before complete coordination. Moreover, the trend of binding energy indicated the increase of the coordinated compound in blending system with *n* increasing before complete coordination.

In addition to enhancing the binding energy of N atoms, the generation of the coordination bond also tuned the energy level and band gap, leading to a new fluorescent center with longer excitation wavelength and emission wavelength. Owing to the weak absorption and emission of BCF after 300 nm (Fig. S10), the influence of free BCF in blending systems is negligible. The absorption (Abs) and photoluminescence (PL) spectra of M1 and M2 with *n* = 0, 0.2, 1, 2 in CF (10^–5 ^mol L^–1^) were depicted in Fig. 1c and 1f. The Abs peak of pure ODPF-Phpy (M1 with *n* = 0) in CF was at 328 nm and the corresponding PL peak was at 401 nm. Pure ODPF-(Phpy)_2_ (M2 with *n* = 0) in CF had an Abs peak at 351 nm and a PL peak at 403 nm. With *n* increasing, a new Abs peak at 371 nm in M1 and at 392 nm in M2 gradually generated, and a new PL peak arose at 480 nm in both M1 and M2. These optical variations can be observed clearly in the PL mapping images (Fig. S11). From the PL mapping images of M1 and M2, the new fluorescent center with a longer excitation wavelength and emission wavelength was gradually enhanced and the original fluorescent center was gradually weakened with *n* increasing, corresponding to the binding energy change of N atoms in the blending system. What’s more, the new fluorescent center has a different fluorescent lifetime from the original one. As shown in Fig. 1d and g, the lifetime of the original fluorescent center (Em = 400 nm) of M1 and M2 was about 1.24 ns and 1.02 ns, respectively. Both the new fluorescent center (Em = 480 nm) in M1 and M2 had a longer lifetime than the original fluorescent center, showing 2.18 and 1.56 ns, respectively. These phenomena suggested that the new fluorescent center was attributed to the coordinated compound. Combining the Abs spectrum with the ultraviolet photoelectron spectroscopy (UPS) data of M1 and M2 (Fig. S12), the HOMO and LUMO of the original compound and coordinated compound (ODPF-Phpy + BCF and ODPF-(Phpy)_2_ + BCF) were calculated in Fig. 1e and 1h. The result showed that the HOMO, LUMO and band gap energy were all lowered after the coordination.

### The photophysical process in coordination blending films

**Fig. 2:**
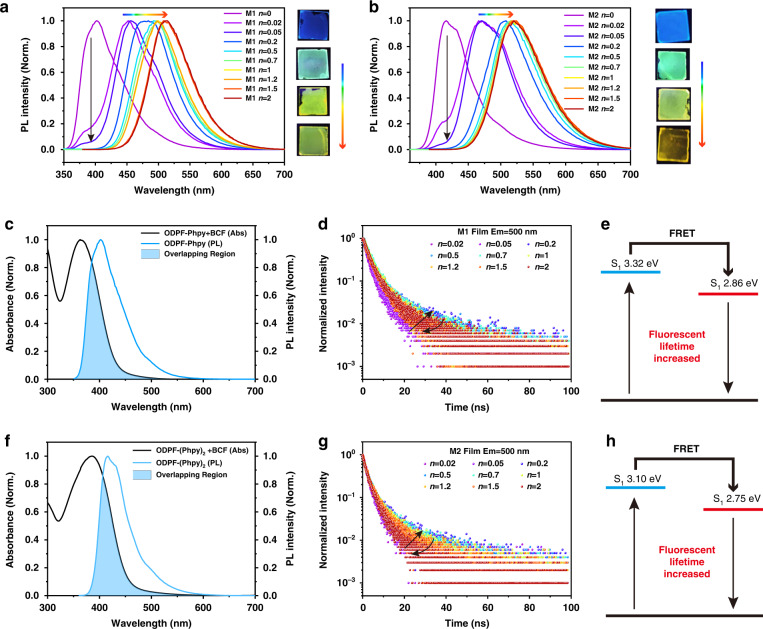
The optical properties of blending films. **a** PL spectra of M1 blending films. (inset: fluorescent photographic images). **b** PL spectra of M2 blending films. (inset: fluorescent photographic images). **c** Abs spectrum of ODPF-Phpy + BCF (coordinated compound, black line) and PL spectrum of ODPF-Phpy (original compound, blue line). The blue area is the overlapping region of the two spectra. **d** Fluorescent lifetime of M1 blending films at 500 nm. **e** The increasing process of the coordinated compound fluorescent lifetime in M1 blending films. **f** Abs spectrum of ODPF-(Phpy)_2_ + BCF (coordinated compound, black line) and PL spectrum of ODPF-(Phpy)_2_ (original compound, blue line). The blue area is the overlapping region of the two spectra. **g** Fluorescent lifetime of M2 blending films at 500 nm. **h** The increasing process of the coordinated compound fluorescent lifetime in M2 blending films

Similar to the molecular state in dilute solution, the original compound and coordinated compound both existed in the blending films before complete coordination. However, the tunable energy level and bandgap induced by supramolecular blending triggered special photoexcitation dynamics of heterogeneous structures in blending films. As shown in Fig. S13, the Abs peak of pure ODPF-Phpy and ODPF-(Phpy)_2_ film was at 330 nm and 354 nm, respectively. The new Abs peak of coordinated compound in M1 and M2 films appeared at 363 nm and 386 nm, respectively. Different from the simple superposition of emission peaks of original and coordinated compounds in solution, the luminescent intensity of the original compounds in blending films decreased sharply after the addition of trace BCF. In the PL spectra of M1 and M2 blending films (Fig. 2a and b), the emission peak of ODPF-Phpy (about 402 nm) and ODPF-(Phpy)_2_ (about 416 nm) declined rapidly as BCF of 2% mole ratio was added, and finally disappeared as *n* = 0.2. Meanwhile, the gradually varying emission peak in PL spectra of blending films (Table S1) implied a complex photophysical process. Here we would explain the photophysical mechanism behind these two phenomena separately.

One of the photophysical processes in blending films was FRET, leading to a sharp attenuation of the PL peak of the original compounds. The PL mapping images of blending films (Figs. S14 and S15) showed the main excitation wavelength changed from about 330 nm to about 370 nm as *n* increased. Especially when *n* = 0.2, the blending film exhibited an excitation of original compound and emission of coordinated compound. This phenomenon implied an energy transfer process from first excited state (*S*_1_) of the original compound to *S*_1_ of the coordinated compound. As displayed in Fig. 2c and f, the large overlapping region between the Abs spectrum of coordinated compound and the PL spectrum of original compound, enabled FRET to occur in blending films. Another consequence of FRET is the change of the fluorescence lifetime of coordinated compound. The fluorescent lifetimes of blending films were listed in Table S2. In the blending films, the fluorescent lifetime of ODPF-Phpy (Em = 410 nm) and ODPF-(Phpy)_2_ (Em = 415 nm) was 1.07 ns and 0.78 ns (Fig. S16), respectively. However, the lifetime of the coordinated compound increased first and then decreased with *n* increasing. For the sake of comparison and explanation, we uniformly compared the fluorescent lifetime at 500 nm. As shown in Fig. 2d and g, when *n* = 0.2, the coordinated compound had the longest fluorescent lifetime, which was 7.50 ns in M1 and 6.46 ns in M2. Correspondingly, the emission peak of original compound finally disappeared when *n* = 0.2, indicating a highest FRET proportion in the blending film. As illustrated in Fig. 2e and h, in blending films before completely coordination, the original compound was first excited to *S*_1_, then the exciton energy transferred to *S*_1_ of coordinated compound via FRET and finally irradiated from *S*_1_ of coordinated compound. This process leads to a longer lifetime than the direct radiative transition of coordinated compound. What’s more, the ratio of FRET to direct excitation affects the fluorescence lifetime. Therefore, the blending film with *n* = 0.2 had the longest fluorescent lifetime owing to the highest FRET proportion. Affected by the emission of the original compound, M1 and M2 with *n* = 0.02 and 0.05 had a shorter fluorescent lifetime. Ultimately, with *n* increasing, FRET results in a rapid disappearance of the emission peak of original compounds and a fluorescence lifetime that first increases and then decreases.

**Fig. 3:**
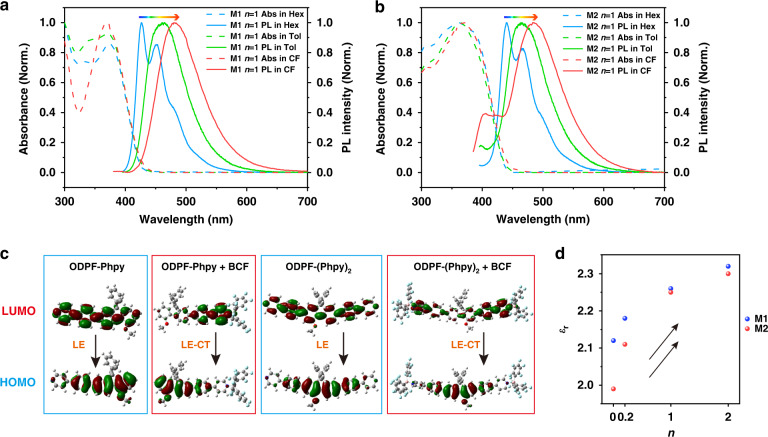
The ICT feature of coordinated compounds. **a** Abs and PL spectra of M1 in Hex, Tol and CF. Concentration: 10^−5 ^mol L^−1^. **b** Abs and PL spectra of M2 in Hex, Tol and CF. Concentration: 10^−5 ^mol L^–1^. **c** The electron density distribution of HOMO and LUMO of original compounds and coordinated compounds. **d** The dielectric constant of blending films with different *n*

Another photophysical process in blending films was the ICT accompanying with the film polarity change, resulting in the red shift of the PL peak of the coordinated compound with *n* increasing. The most important feature of ICT is its strong solvation effect, resulting in a red shift of emission peak with the increase of solvent polarity^3^. As shown in Fig. 3a and b, the coordinated compounds showed a local emission (LE) with clearly fine structure in non-polar solvent hexane (Hex). The four emission peaks of the coordinated compound in M1 in Hex were at 427 nm, 452 nm, 482 nm and 524 nm (Fig. 3a). Similarly, the coordinated compound in M2 had four peaks at 440 nm, 466 nm, 505 nm and 548 nm in PL spectra in Hex (Fig. 3b). Meanwhile, the PL spectra of coordinated compound in toluene (Tol) and CF showed no well-resolved emissive structure and had a clear red shift. The coordinated compound of M1 in Tol and CF had an emission peak at 461 nm and 480 nm, respectively. The coordinated compound of M2 in Tol and CF had an emission peak at 464 nm and 480 nm, respectively. The obvious solvation effect proved the present of ICT in coordinated compounds. On the other hand, the generation of ICT requires a partial delocalization between the HOMO and LUMO of molecules. As presented in Fig. 3c, DFT calculations revealed that the electron density distribution of HOMO and LUMO of the coordinated compounds were severely delocalized, leading to an ICT state of the coordinated compound finally^39^. Different to the LE of ODPF-Phpy and ODPF-(Phpy)_2_, the coordinated compounds displayed a LE-CT emission, which was sensitive to the polarity of molecular environment. The polarity sensitivity of ICT is not only manifested in solution, but also in uniform films. The polarity of the blending film was changed with the variation in component proportions. The dipole moment calculations showed that the coordinated compounds have lager polarity than the original compounds (Table S3). In the blending films, the content of the coordinated compound with larger polarity increasing with *n* enlarged. As displayed in Fig. 3d, the dielectric constant of M1 and M2 blend films gradually increased, suggesting the increasing of film polarity with *n* enlarged. In the blending films, the synergistic effect of the ICT of the coordinated compounds and the change of the film polarity leads to the continuous red shift of the PL peak of coordinated compound with the increase of *n*.

### The photophysical property of the excited state in coordination blending films

**Fig. 4:**
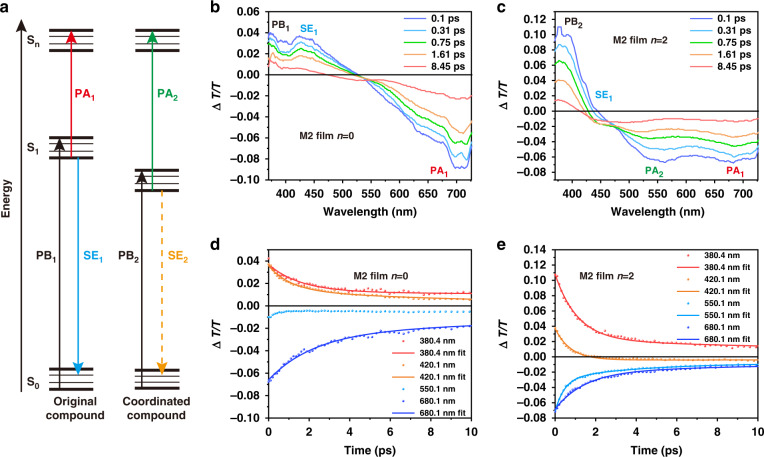
The excited state behaviours of compounds in blending films. **a** TA mechanism of original compound and coordinated compound in blending films. **b** Δ*T/T* spectra of M2 with *n* = 0. **c** Δ*T/T* spectra of M2 with *n* = 2. **d** Δ*T/T* dynamics of M2 with *n* = 0 at different wavelength. **e** Δ*T/T* dynamics of M2 with *n* = 2 at different wavelength

In general, excited state and exciton behavior are seriously modulated by the energy level and complex energy transfer in solid state^40,41^. In coordination blending, the coexisting of the original compound and the coordinated compound endowed the film with two types of excitons. Therefore, two excited state behaviors can be observed in TA spectra. As depicted in Fig. 4a, three main signals, photobleaching (PB), stimulation emission (SE) and photoinduced absorption (PA), can be observed in TA spectra^41^. In order to distinguish the signal of original compound and coordinated compound, we named the three signals of original compound as PB_1_, SE_1_ and PA_1_, and the signals of coordinated compound as PB_2_, SE_2_ and PA_2_. As shown in Fig. S19, for M1 films, the SE_1_ band around 400 nm and the PA_1_ band around 620 nm were ascribed to the original compound exciton. Owing to the limit of spectral region, PB_1_ signal of the original compound exciton cannot be observed in M1 films. For M2 films, a weak PB_1_ signal around 370 nm can be observed. The SE_1_ band was around 430 nm and the PA_1_ band was around 700 nm (Fig. S20). With *n* increasing, the coordinated compound in M1 and M2 both exhibited PB_2_ band around 380 nm and PA_2_ band around 550 nm, and the SE_1_ band of the original compound gradually decreased. Take M2 for example, as *n* increased, the PB_1_ signal was replaced by PB_2_ signal, and the PA_2_ band appeared around 550 nm (Fig. 4b and c). The Δ*T/T* dynamics also showed the replacement process. As shown in Fig. 4d, the decay time of M2 with *n* = 0 at 380.4 nm (PB_1_), 420.1 nm (SE_1_) and 680.1 nm (PA_1_) was 18.82 ps, 4.43 ps and 9.53 ps, respectively. In M2 with *n* = 2, the decay time at 380.4 nm, 420.1 nm, 550 nm (PA_2_) and 680.1 nm was 4.54 ps, 0.77 ps, 3.81 ps and 5.92 ps, respectively (Fig. 4e). It was obvious that the SE_1_ signal decayed rapidly, and the PB_1_ band with long decay time was replaced by PB_2_ band with short decay time. Thus, we verified the two types of excitons and the excited state behaviors in coordination blending films by TA spectroscopy.

### The optical and electrical performance improvement via supramolecular coordination blending

**Fig. 5:**
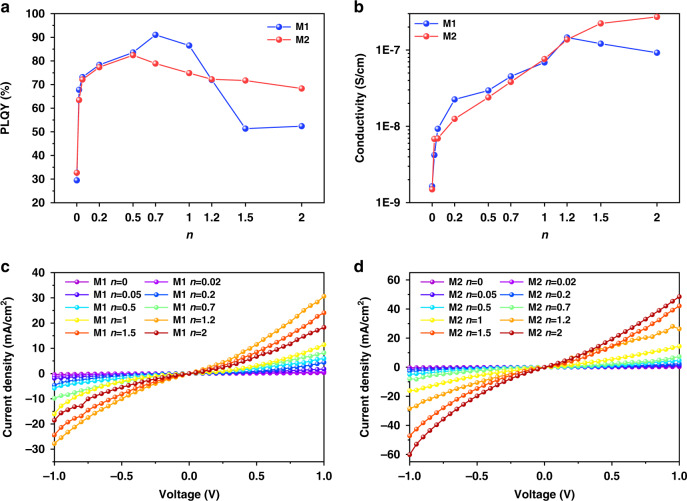
The improvement of blending films in optical and electrical properties. **a** PLQY of blending films. **b** Conductivity of blending films. **c** *J*-*V* curves of M1 blending films. **d** *J*-*V* curves of M2 blending films

For supramolecular coordination blending, the complex photophysical process is also accompanied by the optimization of performance, endowing the materials with a better photoelectric platform. As shown in Fig. 5a and Table S4, ODPF-Phpy and ODPF-(Phpy)_2_ film without blending with BCF showed a PLQY of 29.47% and 32.67%, respectively. As *n* increased, the PLQY of blending films rose rapidly. The maximum PLQY of M1 blending films appeared at *n* = 0.7, which was 91.04%. M2 with *n* = 0.5 had the maximum PLQY of 82.41%. As *n* continued to increase, the PLQY of blending films decreased. The trend of PLQY rising first and then decreasing is caused by the synergistic effect of FRET and ICT. An appropriate proportion of energy transfer can effectively promote the promotion of PLQY^40,42,43^, leading to PLQY increased sharply with small *n* value and reached a maximum of more than 80%. On the other hand, the ICT of coordinated compound would increase the non-radiative transition rate at high concentrations and reduce PLQY^3^. What’s more, the coordination blending also induced the conductivity improvement (Table S4). As shown in Fig. 5b–d, the *J-V* curves showed that ODPF-Phpy and ODPF-(Phpy)_2_ film had a conductivity of about 1.64 × 10^–9 ^S cm^–1^ and 1.50 × 10^–9 ^S cm^–1^. Compared with the original films, the conductivity of blending films can be increased by up to two orders of magnitude. The maximum conductivity of M1 and M2 blending films was 1.46 × 10^–7 ^S cm^–1^ and 2.71 × 10^–7 ^S cm^–1^, respectively. These results indicated that moderate coordination blending ratio can effectively improve the optical and electrical property, providing potential value for organic semiconductors.

By the way, we found that all characterization analysis in this work suggested only one new type exciton for coordinated compounds. Theoretically, owing to the two coordination sites of ODPF-(Phpy)_2_, two coordinated compounds can be generated in M2 blending system. This phenomenon indicated that the two types of coordinated compounds in M2 blending system had the same optical energy level and exhibited the same photophysical property. By analogy, we speculated that no matter how many coordination sites existed on the conjugated backbone, the energy level of multi-coordinated compounds was similar to that of one-coordinated compound. A phenomenon consistent with this inference in conjugated polymers is that a small amount of coordination doping can cause a huge change in band gap. Here we speculated that the most effective use of coordination blending or doping in conjugated polymers was the coordination with the end-functional group.

## Discussion

In summary, we investigated the energy engineering and photoexcitation dynamics of supramolecular N-B coordination blending in fluorene-based light-emitting organic semiconductor films. The binding energy of N-1s showed the N-B coordination bond in the blending films. The absorption and PL spectra in solution exhibited that a new type of exciton with longer excitation wavelength and emission wavelength existed after coordination blending. The two types of exciton have different lifetime that the coordinated compounds have longer fluorescent lifetime than the original compounds. We found the coordinated compound possessed lower HOMO, LUMO and band gap energy than the original compound via UPS. The photophysical process in blending films are complex that the excited state energy transferred from the original compound to the coordinated compound via intermolecular FRET. In addition, the ICT of coordinated compound accompanying with the film polarity change caused the red shift of the PL peak of the coordinated compound with *n* increasing. The excited state behaviors of the coordinated compound in blending system were verified via TA spectroscopy. Finally, the PLQY and conductivity of blending films suggested that moderate coordination blending ratio can effectively improve the optical and electrical performance. We believe the exploration of the internal mechanism of coordination blending can provide potential application and development for organic electronics.

## Materials and methods

### Chemicals

All reagents were purchased from Sigma-Aldrich, Merck and Alfa Aesar, and used as received unless stated otherwise. Anhydrous THF, CF, Tol and Hex (HPLC grade) were collected from Solvent Purification Systems (Innovative Technology, Inc.). Anhydrous chloroform was pre-dried over molecular sieves.

### Structure and heat characterization

NMR spectra were recorded on a Bruker 400 MHz spectrometer in CDCl_3_ with tetramethylsilane (TMS) as the interval standard. Matrix Assisted Laser Desorption Ionization (coupled to a Time-Of-Flight analyzer) experiments (MALDI-TOF) was recorded on a Shimadzu GCMS 2010 PLUS. Thermogravimetric analyses (TGA) measurements were conducted by a Shimadzu DTG-60H under a heating rate of 10^o^C min^–1^ and a nitrogen flow rate of 50 cm^3^ min^–1^. Differential scanning calorimetry (DSC) measurements were performed using a Shimadzu Instruments DSC-60A and DSC data were collected from 30 to 200 ^o^C at a rate of 10 ^o^C min^–1^ under N_2_ flow.

### Cyclic voltammetric (CV) test

CV studies were conducted using an CHI660C Electrochemical Work station in a typical three-electrode cell with a platinum sheet working electrode, a platinum wire counter electrode, and a silver/silver nitrate (Ag/Ag+) reference electrode. All electrochemical experiments were carried out under a nitrogen atmosphere at room temperature in an electrolyte solution of 0.1 M tetrabutylammonium hexafluorophosphate (*n*-Bu_4_NPF_6_) in CH_3_CN at a sweeping rate of 0.1 V s^–1^. According to the redox onset potentials of the CV measurements, the HOMO/LUMO energy levels of the materials are estimated based on the reference energy level of ferrocene (4.8 eV below the vacuum): HOMO/LUMO = −[*E*_onset_ –*E*_(Fc/Fc+)_+ 4.8] eV.

### Optical properties

UV-vis absorption spectra were measured with a Shimadzu UV-3600 spectrometer at room temperature, and PL spectra and excitation-emission 2D spectra (PL Mapping) were recorded on a Hitachi F-4600 luminescence spectrometer. Nanosecond time-resolved studies, PLQY and variable temperature PL spectra were performed with an Edinburgh FLS 980 time-correlated single photon-counting (TCSPC). Ultraviolet Photoelectron Spectroscopy (UPS) was performed by PHI 5000 VersaProbe III with He I source (21.22 eV) under an applied negative bias of 0 V and –5.0 V.

### X-ray photoelectron spectroscopy (XPS)

XPS was conducted on a Thermo Scientific^TM^ K-Alpha^TM+^ spectrometer equipped with a monochromatic Al Kα X-ray source (1486.6 eV) operating at 100 W. Samples were analyzed under vacuum (*P* < 10^−8^ mbar) with a pass energy of 150 eV (survey scans) or 50 eV (high-resolution scans). All peaks would be calibrated with C1s peak binding energy at 284.8 eV for adventitious carbon.

### Computational methods

The geometries of the ground and first singlet excited states were fully optimized by density functional theory (DFT) and time dependent DFT (TD-DFT) at the CAM-B3LYP/6-31 G* level, respectively^39^.

### Dielectric constant test

The device structure for capacitance measurement was Cu/Material Layer/Si. Capacitance of the material layers were carried out by HIOHI IM3533-01. The dielectric constant was calculated by $C_i = \frac{{\xi _0K}}{d}$, where $C_i$ is the capacitance, $\xi _0$ is the vacuum permittivity and *d* is the film thickness.

### Femtosecond resolved TA spectroscopy

The pump beam was generated from a regenerative amplified Ti:sapphire laser system from Coherent (800 nm, 100 fs, 6 Mj per pulse, and 1 kHz repetition rate). The 800 nm output pulse from the regenerative amplifier was split into two parts with a beam splitter. The reflected part was used to pump a TOPAS Optical Parametric Amplifier (OPA) which generates a wavelength-tunable laser pulse from 250 to 2.5 mm as the pump beam. The transmitted 800 nm beam was attenuated with a neutral density filter and focused into a rotating CaF_2_ disk to generate a white light continuum (WLC) from 350 to 800 nm used for the probe beam. The probe beam was focused with an Al parabolic reflector onto the sample. After the sample, the probe beam was collimated and then focused into a fiber-coupled spectrometer and detected at a frequency of 1 kHz. The intensity of the pump pulse used in the experiment was controlled by a variable neutral-density filter wheel. The delay between the pump and probe pulses was controlled by a motorized delay stage. The pump pulses were chopped by a synchronized chopper at 500 Hz.

### Conductivity measurement

The conductivity measurement was performed on the device with structure of ITO/Blending Film/Al. The measuring voltage was from −1 V to 1 V, which was supplied by Keithley 2400. The conductivity was calculated by $\sigma= \frac{{Id}}{{VA}}$, where *I* is the current, *V* is the voltage, *A* is the device area and *d* is the film thickness.

## Supplementary information

Supporting Infomation

## Data Availability

If data are in an archive, include the accession number or a placeholder for it. Also include any materials that must be obtained through an MTA.
