# Supplementary material for: Photoexcitation dynamics and energy engineering in supramolecular doping of organic conjugated molecules
Source: Light Sci Appl. 2023 Jan 31;12:30. doi: 10.1038/s41377-022-01062-6 (PMC9889348; doi:10.1038/s41377-022-01062-6)
Supplement: Supplementary file 1 — Supporting Infomation [file 41377_2022_1062_MOESM1_ESM.docx]

Supplementary Information for

**Photoexcitation dynamics and energy engineering in supramolecular doping of organic conjugated molecules**

Xiang An,^1^† Chuanxin Wei,^2^† Lubing Bai,^1^† Jun Zhou,^3^ Le Wang,^2^ Yamin Han,^1^ Lili Sun,^1^ Jinyi Lin,^1,4^* Heyuan Liu,^3^ Jiewei Li,^1^ Man Xu,^2^ Haifeng Ling,^2^ Linghai Xie,^2,4^ and Wei Huang^1,4^*

^1^Key Laboratory of Flexible Electronics (KLOFE) & Institute of Advanced Materials (IAM), Nanjing Tech University (NanjingTech), 30 South Puzhu Road, Nanjing 211816, China.

^2^State Key Laboratory of Organic Electronics and Information Displays & Institute of Advanced Materials (IAM), Nanjing University of Posts & Telecommunications, 9 Wenyuan Road, Nanjing 210023, China.

^3^College of Science and Institute of New Energy, China University of Petroleum (East China), Qingdao 266580, China.

^4^Shaanxi Institute of Flexible Electronics (SIFE), Northwestern Polytechnical University (NPU), 127 West Youyi Road, Xi'an 710072, Shaanxi, China.

†Xiang An, Chuanxin Wei and Lubing Bai are contributed equally to this work.

*Correspondence: iamjylin@njtech.edu.cn (J.Y. Lin); wei-huang@njtech.edu.cn (W. Huang).

**Supplementary Information**

**Section S1. Synthesis of ODPF-Phpy and ODPF-(Phpy)_2_.**


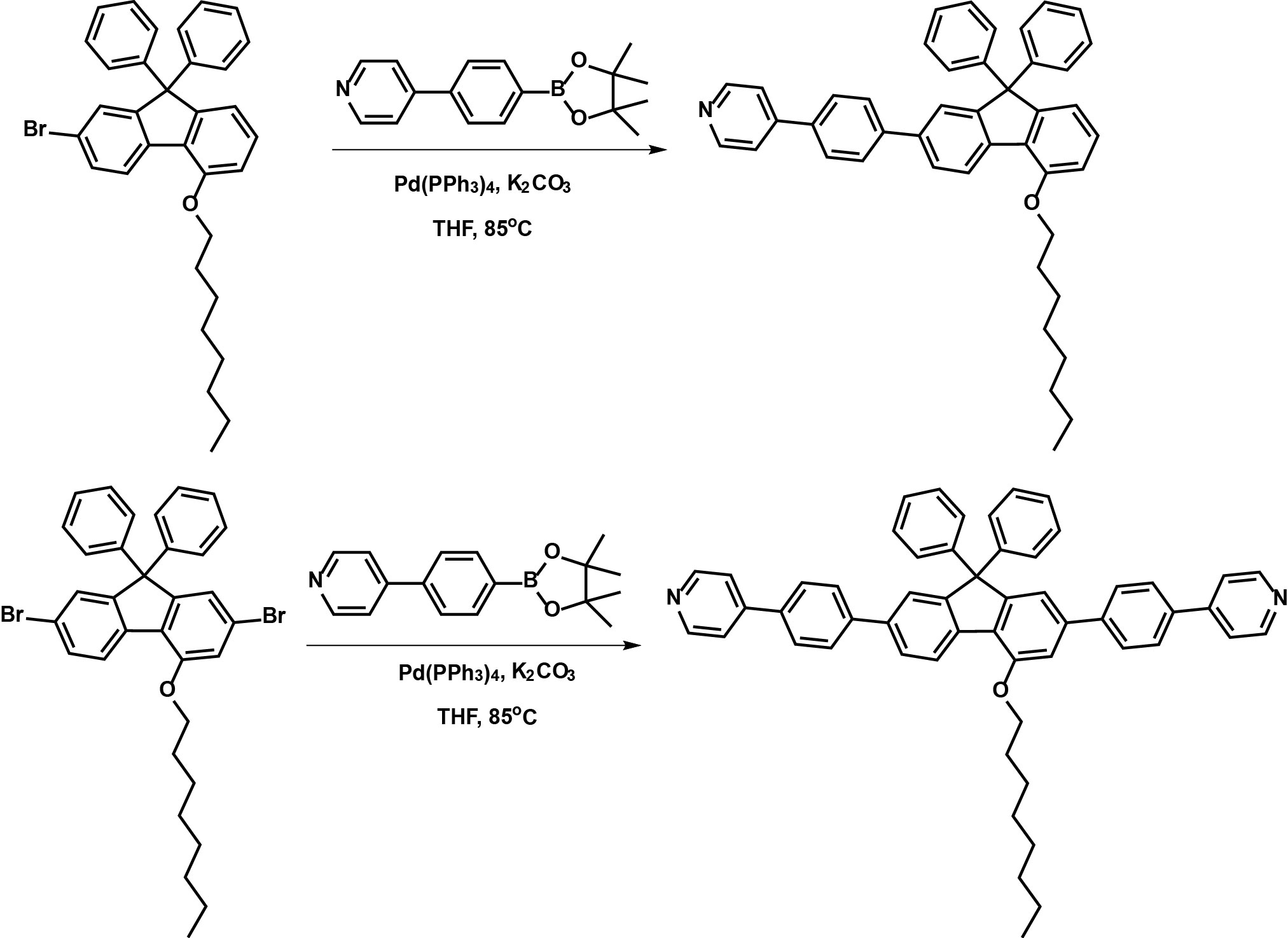


**Scheme S1.** Synthesis and molecular structure of ODPF-Phpy and ODPF-(Phpy)_2_.

**4-(4-(5-(octyloxy)-9,9-diphenyl-9H-fluoren-2-yl)phenyl)pyridine (ODPF-Phpy).**

Under N_2_ condition, 2-bromo-5-(octyloxy)-9,9-diphenyl-9H-fluorene (0.53 g, 1.01 mmol), 4-(4-(4,4,5,5-tetramethyl-1,3,2-dioxaborolan-2-yl)phenyl)pyridine (0.85 g, 3.02 mmol) and tetrakis(triphenylphosphine)palladium(0) (0.09 g, 0.10 mmol) were added into tetrahydrofuran (THF). After adding 2 mol L^-1^ aqueous solution of K_2_CO_3_ (3 mL) to the mixture, the mixture was stirred at 85^o^C for 24 hours. Then the mixture was separated by suction filtration using Buchner funnel and washed by THF for several times. The filter liquor was dried with Na_2_SO_4_, and the solution was evaporated in vacuum. ODPF-Phpy (80%) was obtained from chromatography using an eluent of ethyl acetate (EA) with several drops of triethylamine. ^1^H-NMR (400 MHz, Chloroform-*d*) δ 8.64 (d, *J* = 16.8 Hz, 2H), 8.23 (s, 1H), 7.68 (s, 6H), 7.53 (s, 2H), 7.23 (d, *J* = 14.0 Hz, 11H), 7.00 (d, *J* = 12.4 Hz, 1H), 6.86 (d, *J* = 8.8 Hz, 1H), 4.18 (s, 2H), 2.05 – 1.94 (m, 2H), 1.64 (s, 2H), 1.44 (d, *J* = 9.4 Hz, 2H), 1.33 (s, 6H), 0.91 (s, 3H). ^13^C-NMR (101 MHz, Chloroform-*d*) δ 155.59, 153.39, 151.55, 150.29, 148.00, 145.98, 142.36, 139.54, 138.54, 136.65, 135.61, 128.99, 128.35 (d, *J* = 1.9 Hz), 127.88, 127.38, 126.68 (d, *J* = 14.3 Hz), 126.38, 124.49, 124.26, 121.54, 118.28, 110.07, 68.22, 65.87, 31.95, 29.91 – 29.28 (m), 26.39, 22.81, 14.27. MALDI-TOF-MS: 599.381 (m/z).

**4,4'-((4-(octyloxy)-9,9-diphenyl-9H-fluorene-2,7-diyl)bis(4,1-phenylene))dipyridine (ODPF-(Phpy)_2_).**

Under N_2_ condition, 2,7-dibromo-4-(octyloxy)-9,9-diphenyl-9H-fluorene (0.61 g, 1.01 mmol), (4,4,5,5-tetramethyl-1,3,2-dioxaborolan-2-yl)phenyl)pyridine (0.85 g, 3.02 mmol) and tetrakis(triphenylphosphine)palladium(0) (0.09 g, 0.10 mmol) were added into THF. After adding 2mol L^-1^ aqueous solution of K_2_CO_3_ (3 mL) to the mixture, the mixture was stirred at 85^o^C for 24 hours. Then the mixture was separated by suction filtration using Buchner funnel and washed by THF for several times. The filter liquor was dried with Na_2_SO_4_, and the solution was evaporated in vacuum. ODPF-(Phpy)_2_ (76%) was obtained from chromatography using an eluent of EA with several drops of triethylamine. ^1^H-NMR (400 MHz, Chloroform-*d*) δ 8.68 – 8.65 (m, 4H), 8.27 (d, *J* = 8.0 Hz, 1H), 7.77 (d, *J* = 0.7 Hz, 1H), 7.70 – 7.66 (m, 10H), 7.55 – 7.52 (m, 4H), 7.34 – 7.30 (m, 4H), 7.28 (d, *J* = 1.3 Hz, 2H), 7.25 – 7.22 (m, 4H), 7.13 (d, *J* = 1.2 Hz, 1H), 4.28 (t, *J* = 6.4 Hz, 2H), 2.04 (dt, *J* = 14.5, 6.5 Hz, 2H), 1.64 (s, 2H), 1.52 – 1.44 (m, 2H), 1.43 – 1.37 (m, 2H), 1.36 – 1.32 (m, 4H), 0.93 – 0.89 (m, 3H). ^13^C-NMR (101 MHz, Chloroform-*d*) δ 155.79, 154.05, 151.85, 150.61 – 150.24 (m), 147.90 (d, *J* = 6.9 Hz), 145.85, 142.31 (d, *J* = 10.6 Hz), 141.27, 139.04, 138.79, 137.06, 136.76, 128.43 (d, *J* = 6.7 Hz), 128.19 – 127.77 (m), 127.77 – 127.28 (m), 126.81 (d, *J* = 11.2 Hz), 124.42 (d, *J* = 19.8 Hz), 121.55, 117.24, 109.27, 68.38, 66.05, 31.95, 29.65 – 29.26 (m), 26.40, 22.81, 14.27. MALDI-TOF-MS: 752.519 (m/z).

**Section S2. Structure information and heat property of ODPF-Phpy, ODPF-(Phpy)_2_ and BCF.**


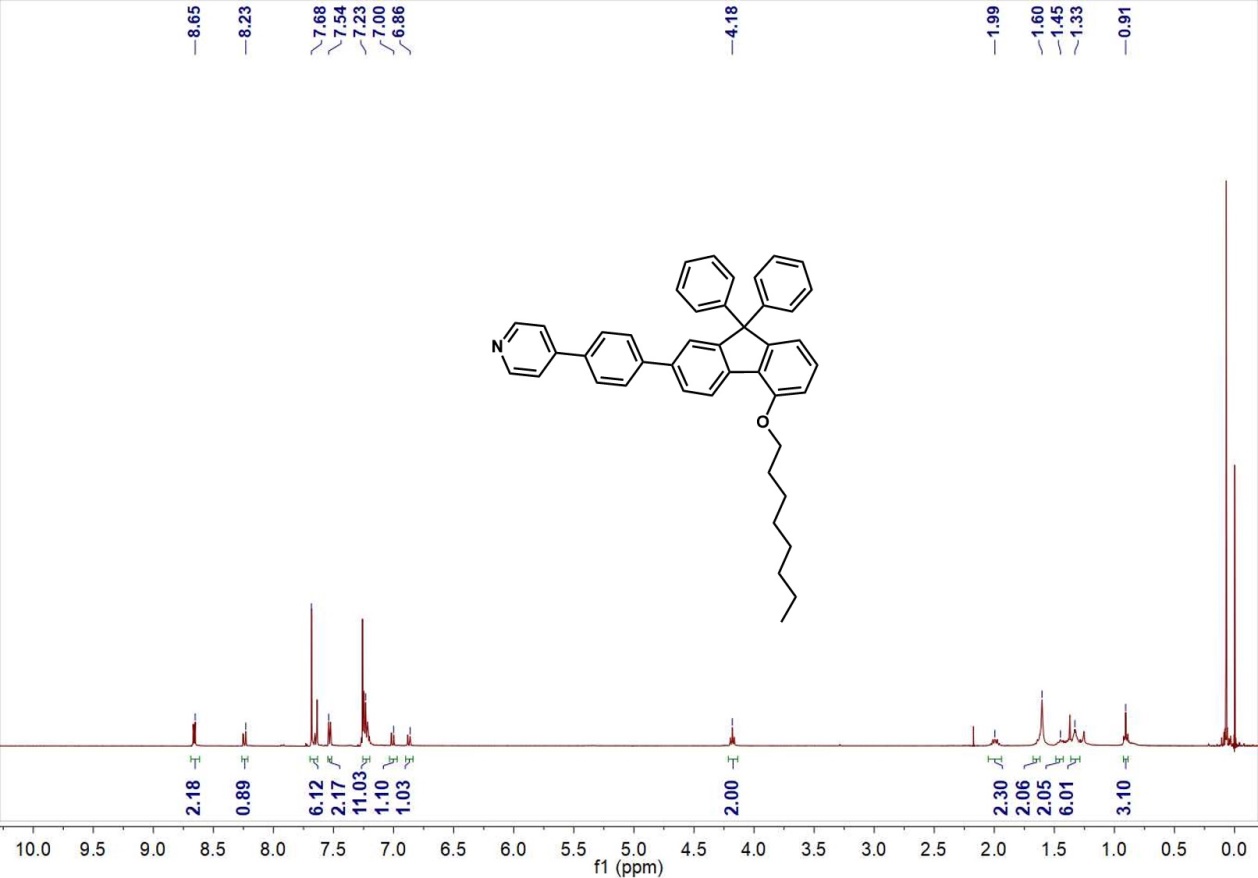


**Figure S1.** ^1^H-NMR of ODPF-Phpy in CDCl_3_.


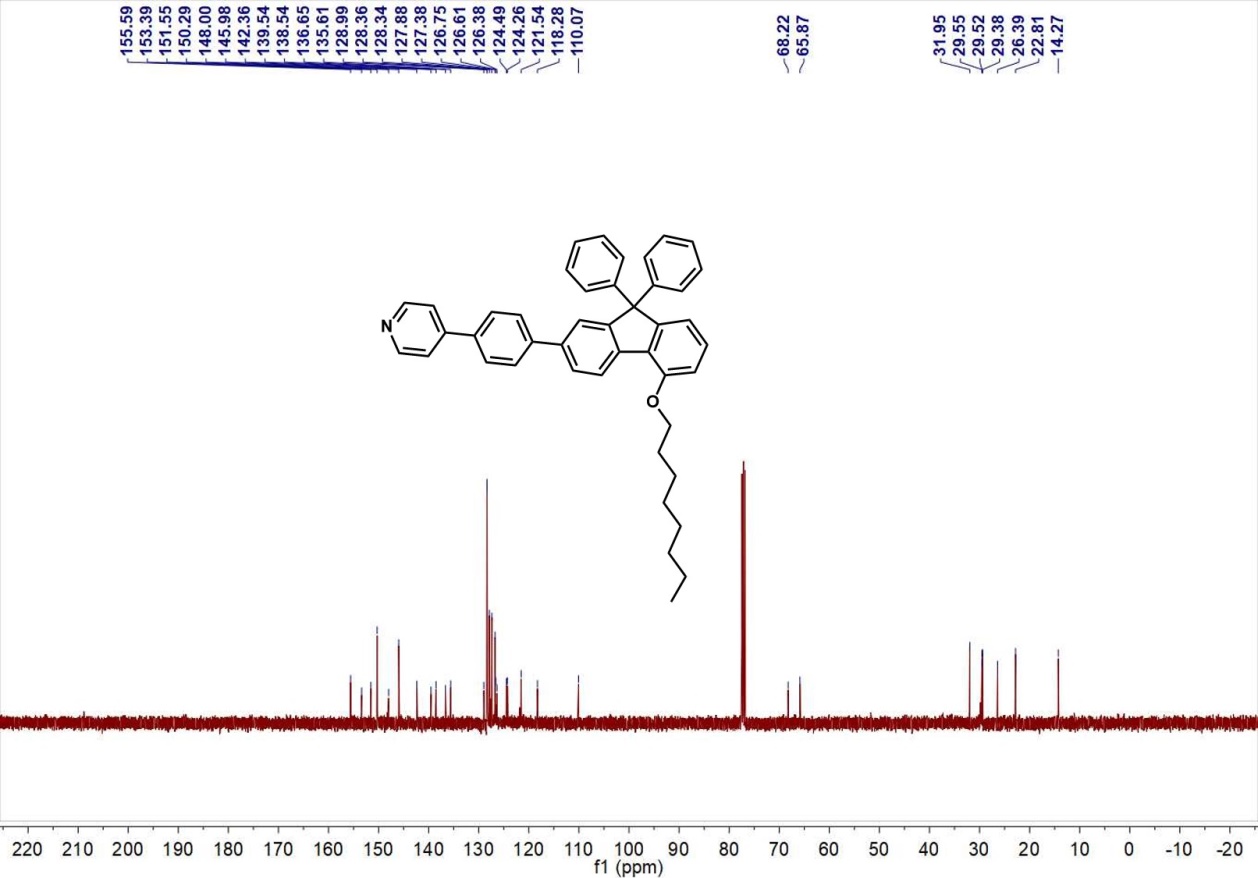


**Figure S2.** ^13^C-NMR of ODPF-Phpy in CDCl_3_.


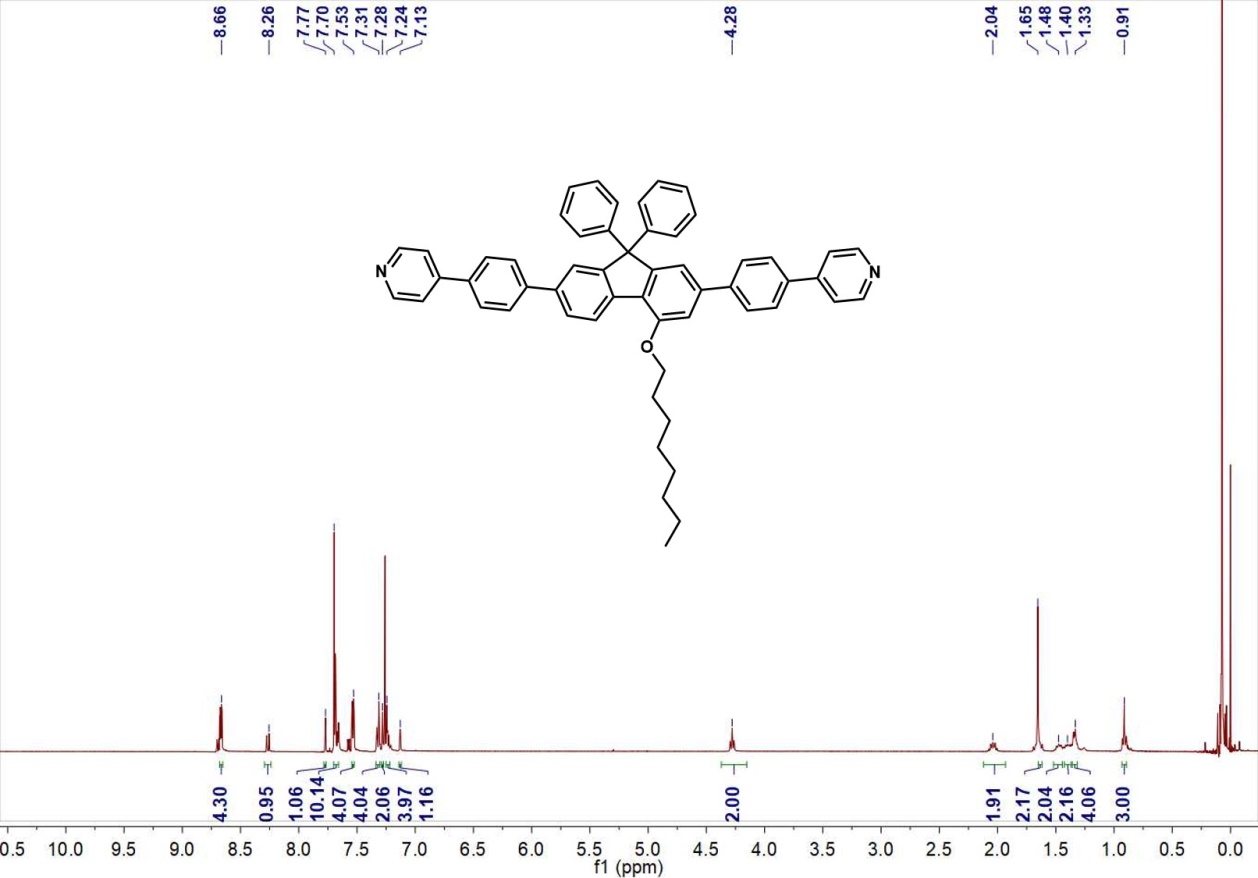


**Figure S3.** ^1^H-NMR of ODPF-(Phpy)_2_ in CDCl_3_.


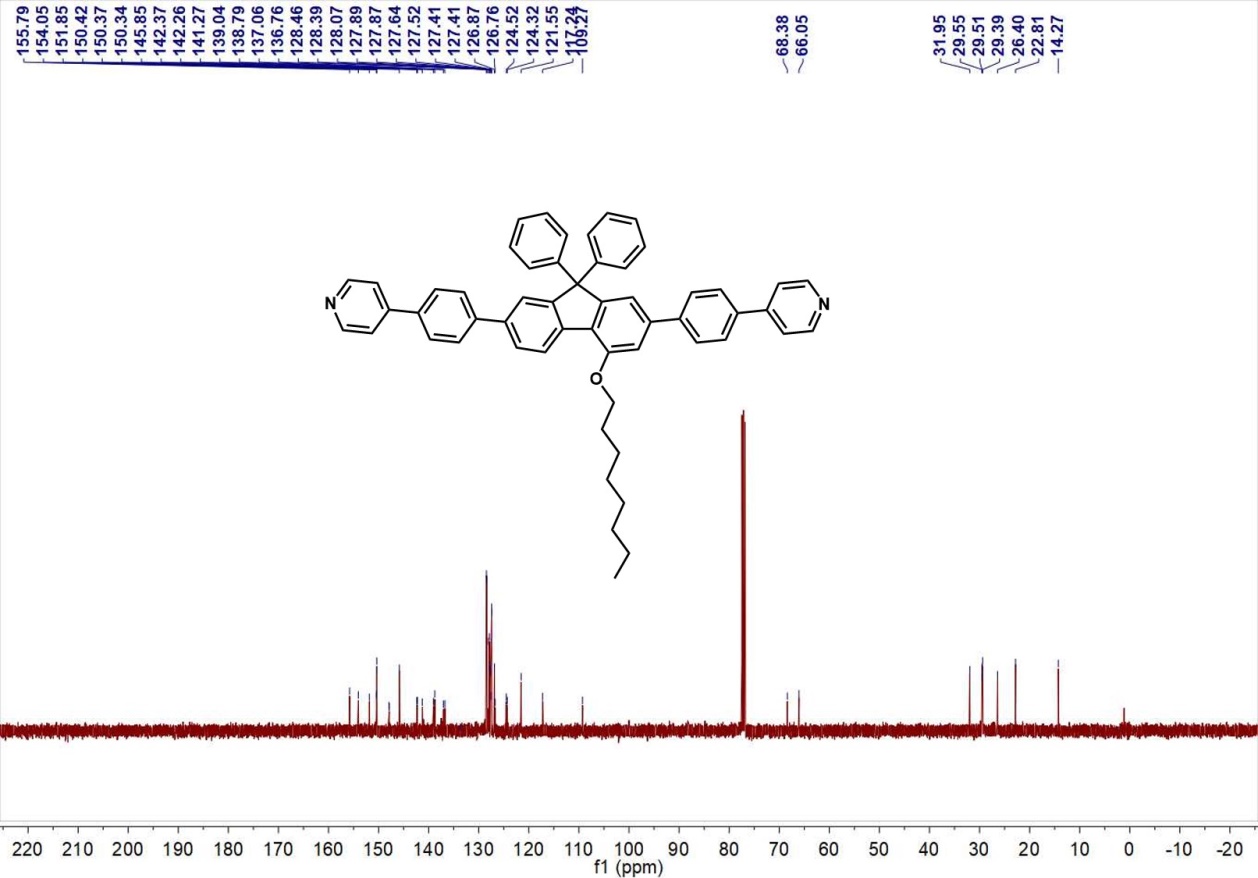


**Figure S4.** ^13^C-NMR of ODPF-(Phpy)_2_ in CDCl_3_.

**Figure S5.** MALDI-TOF-MS of (a) ODPF-Phpy and (b) ODPF-(Phpy)_2_.


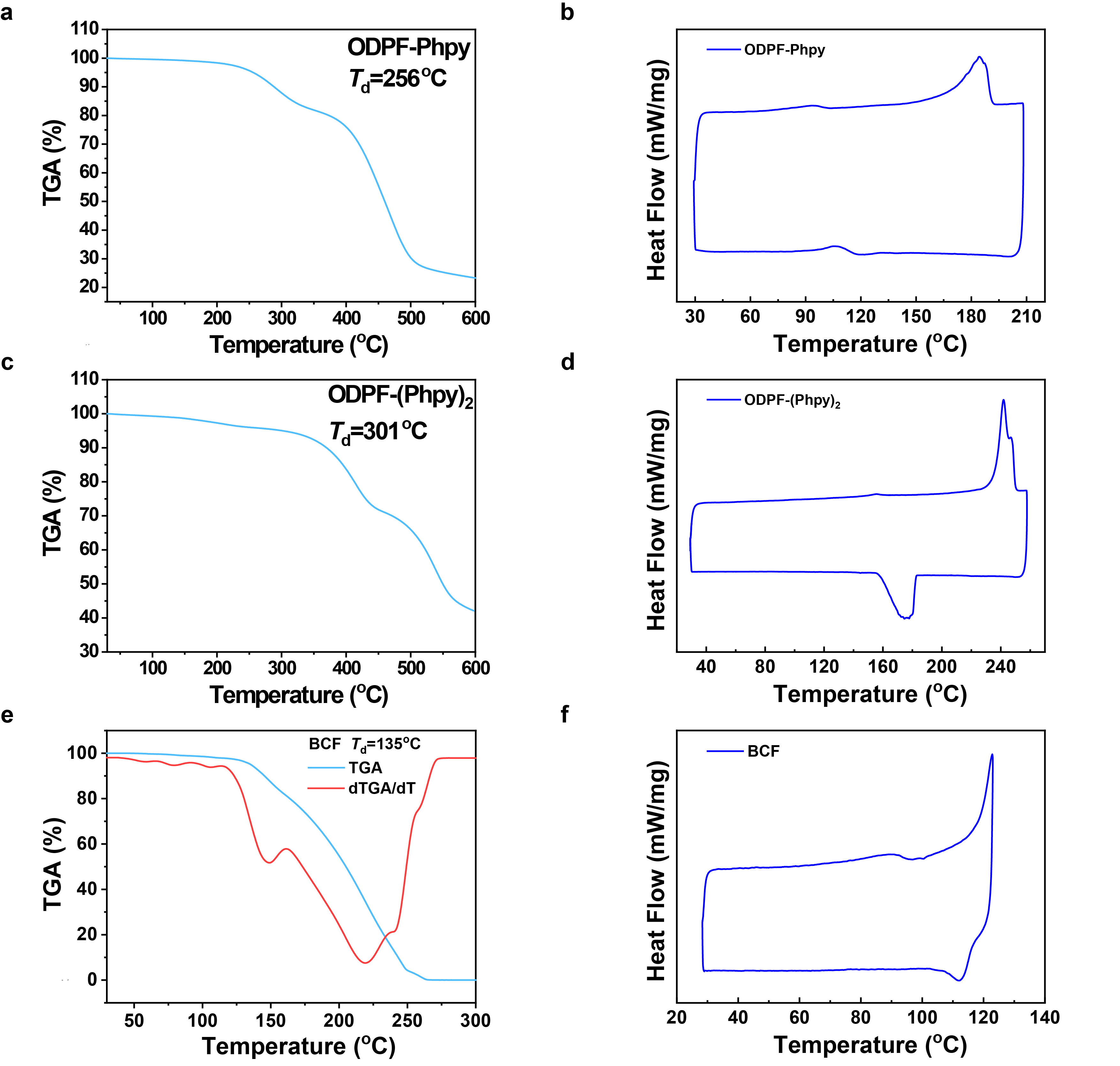


**Figure S6.** (a) TGA of ODPF-Phpy. (b) DSC curve of ODPF-Phpy. (c) TGA of ODPF-(Phpy)_2_. (d) DSC curve of ODPF-(Phpy)_2_. (e) TGA of BCF. (b) DSC curve of BCF.

**Section S3. XPS data of BCF, M1 and M2 films.**

**Figure S7.** The XPS data of BCF film. (a) XPS C-1s region for BCF film. (b) XPS F-1s region for BCF film. (c) XPS B-1s region for BCF film.

**Figure S8.** The XPS data of M1 blending films. (a) XPS N-1s region for M1 films. (b) XPS C-1s region for M1 films. (c) XPS F-1s region for M1 films. (d) XPS B-1s region for M1 films.

**Figure S9.** The XPS data of M2 blending films. (a) XPS N-1s region for M2 films. (b) XPS C-1s region for M2 films. (c) XPS F-1s region for M2 films. (d) XPS B-1s region for M2 films.

**Section S4. Photophysical properties of M1 and M2 blending systems.**

**Figure S10.** (a) Abs spectrum of BCF film. (b) PL mapping image of BCF film. (c) Cyclic voltammetry (CV) curves of BCF. (d) CV curves of ODPF-Phpy.

The absorbance and PL intensity of BCF are too weak. It is unfortunate that we cannot achieve the Abs spectrum of BCF dilute solution and the PL spectra of BCF film and dilute solution. BCF is nearly nonluminous so we only achieved weak signals in PL mapping image although the slits were set to maximum. Meanwhile, due to the difficulty of obtaining uniform BCF film, it was a pity that we cannot obtain valid UPS data of BCF. In order to compare the energy level difference between BCF and the host materials, we obtained approximate HOMO and LUMO by CV curves. The HOMO and LUMO of materials can be calculated by $HOMO=-(E_{\mathrm{Oxi}}-E_{FC/\mathrm{FC}^{+}})-4.8$ and $LUMO=-(E_{\mathrm{Red}}-E_{FC/\mathrm{FC}^{+}})-4.8$. Therefore, the HOMO and LUMO of BCF was calculated as -6.48 eV and -2.53 eV. The HOMO and LUMO of ODPF-Phpy was calculated as -5.90 eV and -2.49 eV. This result showed that BCF has a deep HOMO energy level and a large bandgap of about 3.95 eV. Therefore, in terms of electronic structure, BCF cannot directly affect on the photophysical properties of blending system.


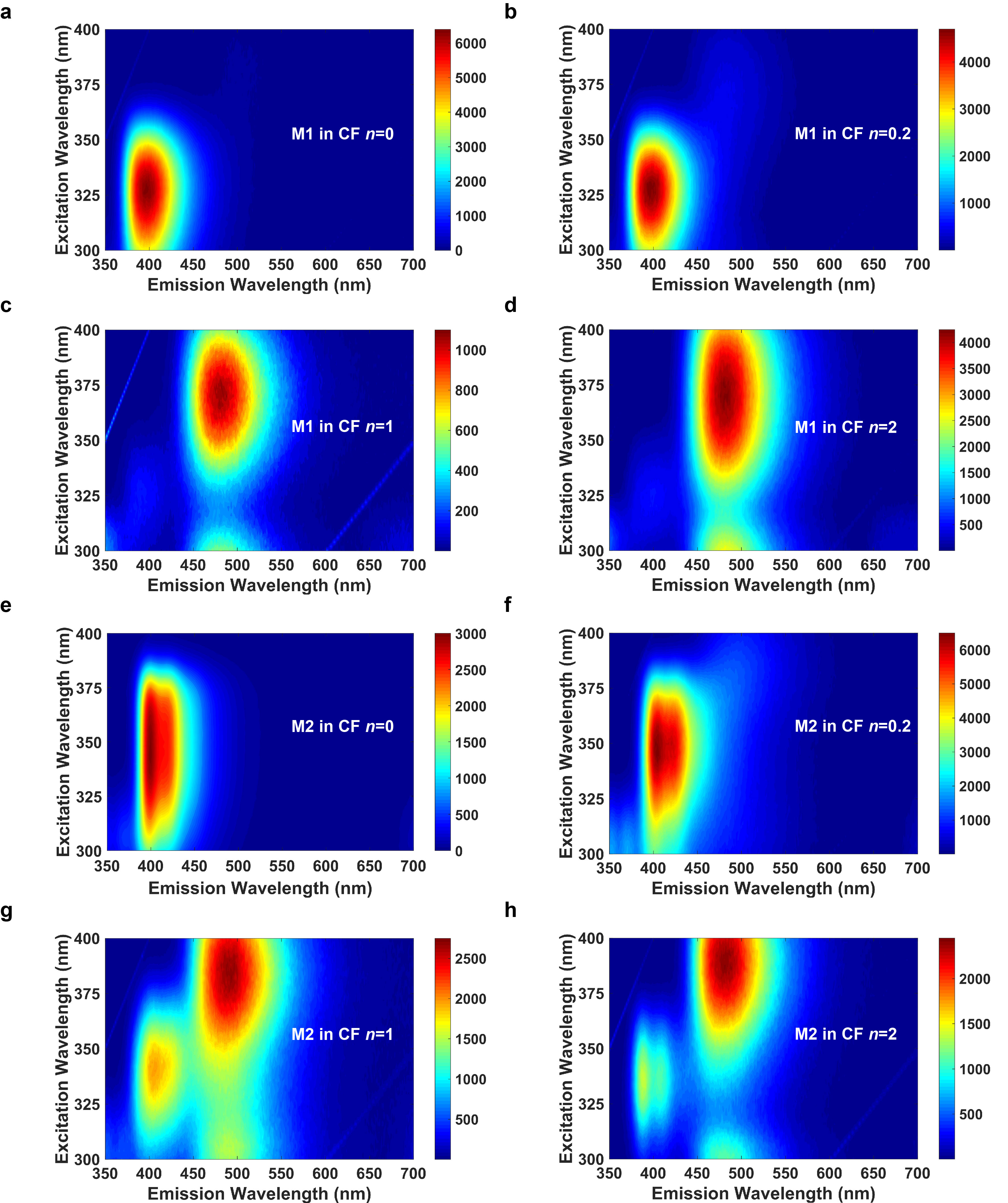


**Figure S11.** (a)-(d) PL mapping images of M1 with *n=*0, 0.2, 1 and 2 in CF. (e)-(h) PL mapping images of M2 with *n=*0, 0.2, 1 and 2 in CF.

**Figure S12.** (a)(b) UPS spectra of M1 blending films with *n=*0, 1 and 2. The work function was 4.14 eV, 4.08 eV and 4.21 eV, respectively. (c)(d) UPS spectra of M2 blending films with *n=*0, 1 and 2. The work function was 4.21 eV, 4.04 eV and 4.07 eV, respectively.

**Figure S13.** (a) Abs and spectrum of M1 blending films. (c) Abs spectrum of M2 blending films.


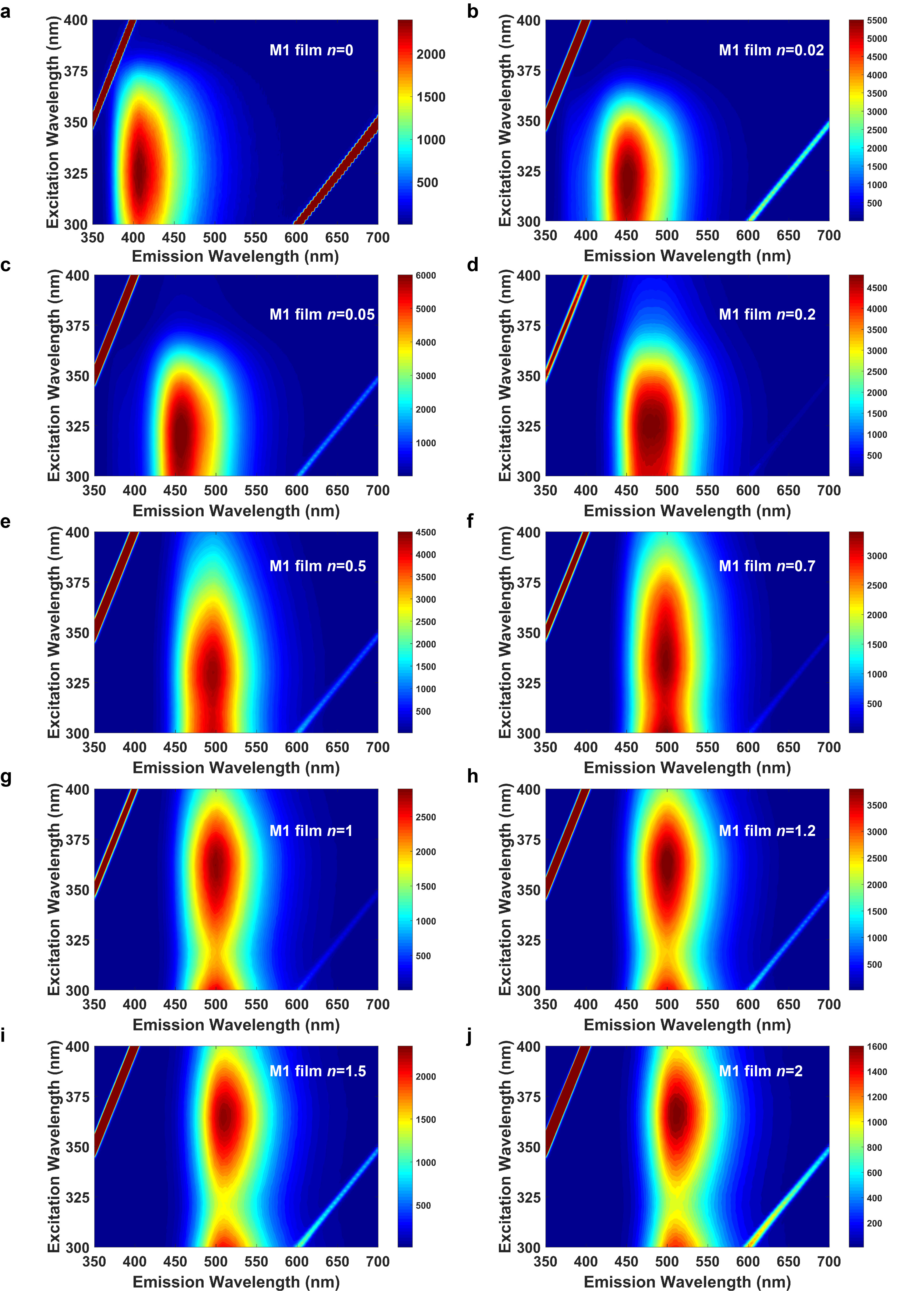


**Figure S14.** PL mapping image of M1 films with *n*=0, 0.02, 0.05, 0.2, 0.5, 0.7, 1, 1.2, 1.5 and 2.


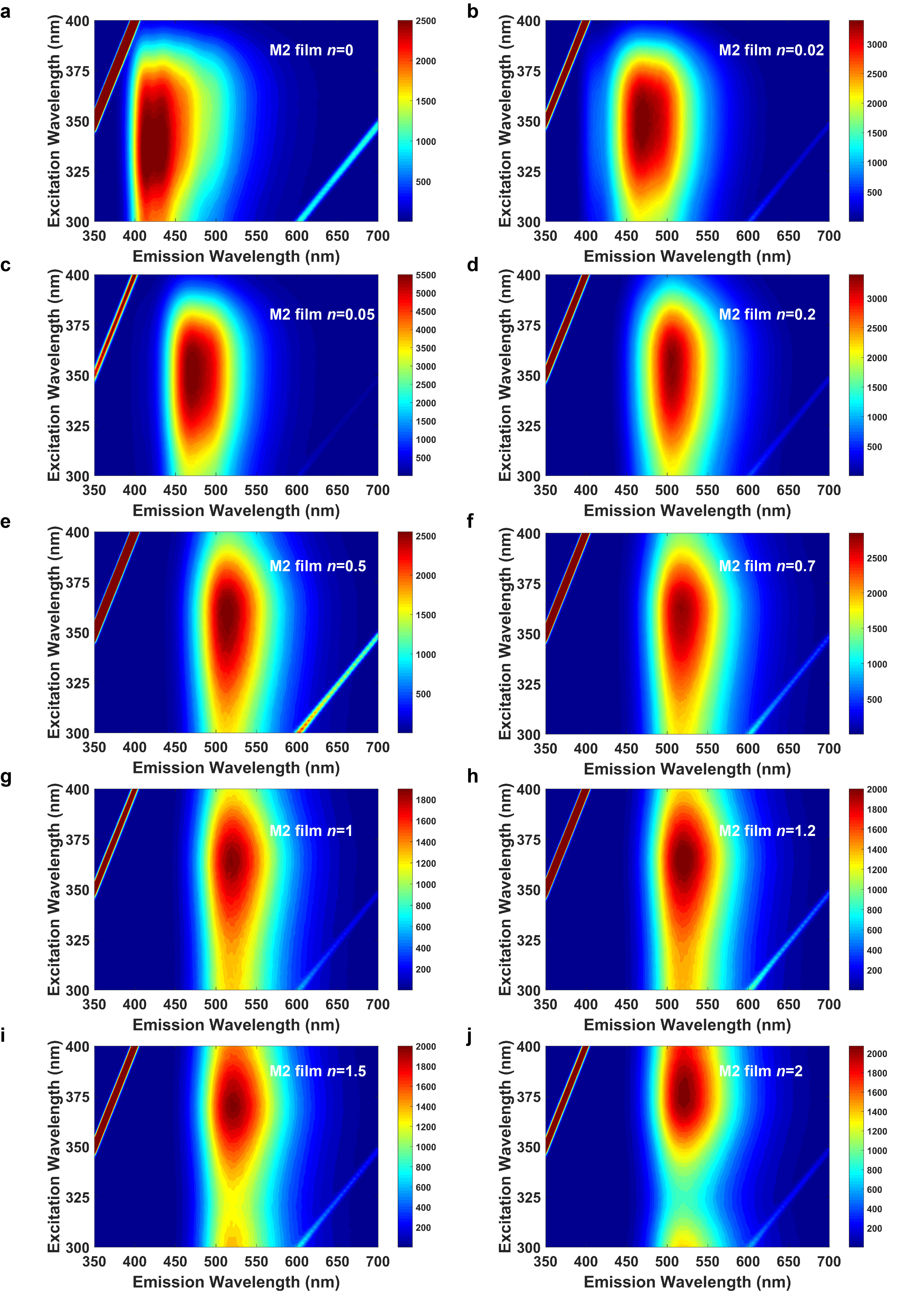


**Figure S15.** PL mapping image of M2 films with *n*=0, 0.02, 0.05, 0.2, 0.5, 0.7, 1, 1.2, 1.5 and 2.

**Table S1.** The main PL peak of blending films.

| *n* | Emission Wavelength (nm) | |
| --- | --- | --- |
|  | M1 | M2 |
| 0.02  0.05  0.2  0.5  0.7  1  1.2  1.5  2 | 452  456  484  492  496  498  500  512  514 | 466  470  504  512  514  516  518  520  520 |

**Figure S16.** (a) Fluorescent lifetime of M1 with *n*=0, 0.02 and 0.05 at 410 nm. (a) Fluorescent lifetime of M2 with *n*=0, 0.02 and 0.05 at 415 nm.

**Table S2.** Fluorescent lifetime of M1 and M2 blending films.

| *n* | *τ*_M1_ (ns) | *τ*_M2_ (ns) |
| --- | --- | --- |
| 0  0.02  0.05  0.2  0.5  0.7  1  1.2  1.5  2 | 1.07*^a^*  2.54*^a^*, 5.19*^c^*  2.50*^a^*, 4.56*^c^*  7.50*^c^*  6.99*^c^*  6.58*^c^*  5.84*^c^*  5.75*^c^*  5.69*^c^*  5.79*^c^* | 0.78*^b^*  1.29*^b^*, 4.47*^c^*  1.42*^b^*, 4.11*^c^*  6.46*^c^*  5.92*^c^*  5.84*^c^*  5.27*^c^*  5.00*^c^*  4.65*^c^*  4.06*^c^* |

*^a^* Em=410 nm. *^b^* Em=415 nm. *^c^* Em=500 nm.


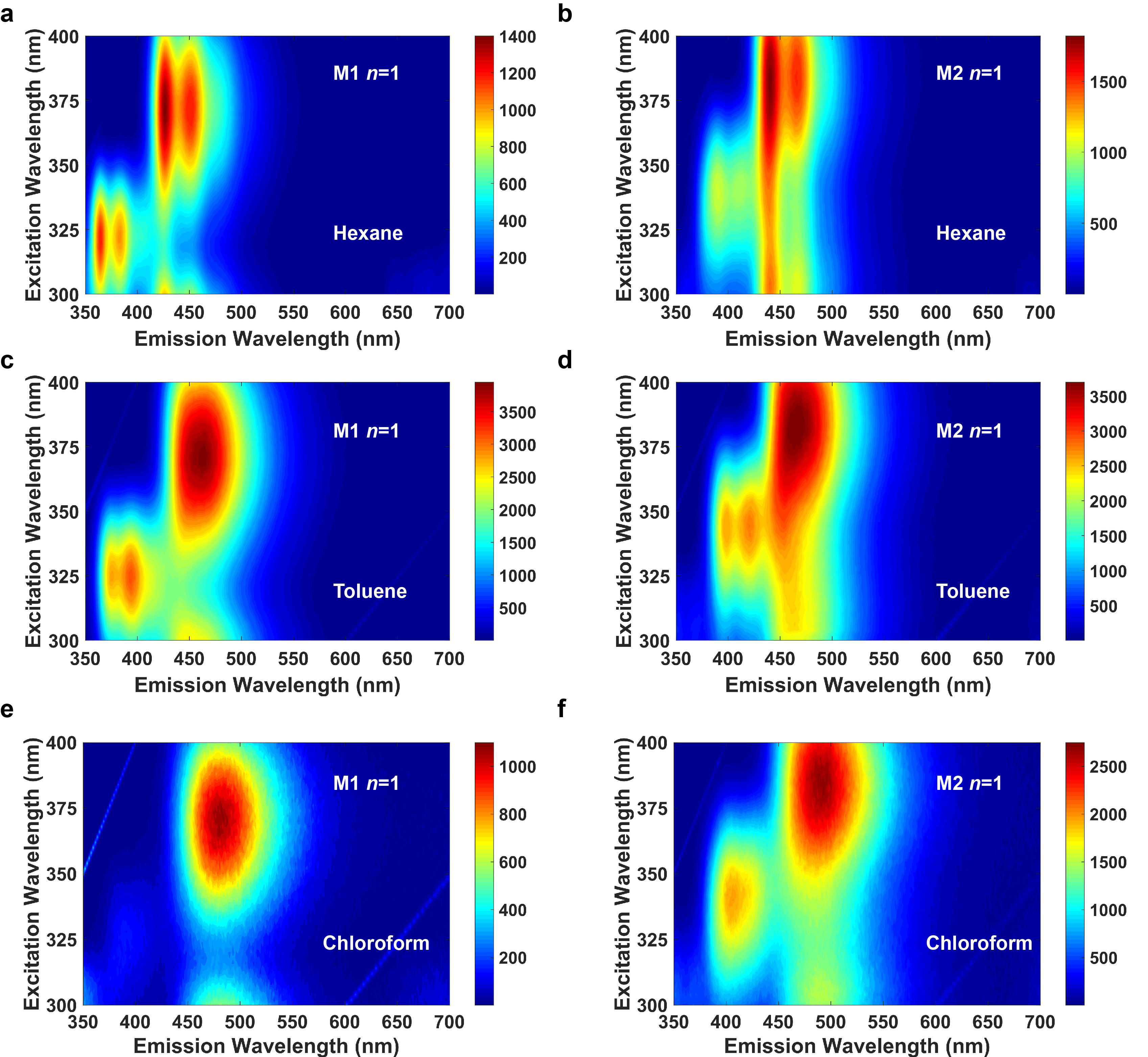


**Figure S17.** (a) PL mapping image of M1 with *n=*1 in Hex. (b) PL mapping image of M2 with *n=*1 in Hex. (c) PL mapping image of M1 with *n=*1 in Tol. (d) PL mapping image of M2 with *n=*1 in Tol. (e) PL mapping image of M1 with *n=*1 in CF. (f) PL mapping image of M2 with *n=*1 in CF.

**Figure S18**. Abs and PL spectra of (a) ODPF-Phpy and (b) ODPF-(Phpy)_2_ in Hex, Tol and CF.

**Table S3.** Results of dipole moment calculations of ODPF-Phpy, ODPF-Phpy+BCF, ODPF-(Phpy)_2_ and ODPF-(Phpy)_2_+BCF.

|  | ODPF-Phpy | ODPF-Phpy+BCF | ODPF-(Phpy)_2_ | ODPF-(Phpy)_2_+BCF |
| --- | --- | --- | --- | --- |
| Dipole moment*^a^* | 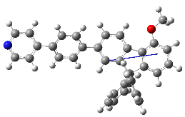  5.07 D | 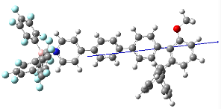  14.89 D | 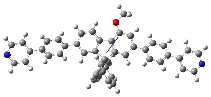  2.66 D | 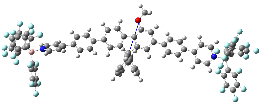  5.37 D |

*^a^* Calculated by DFT method.

The PL spectra of ODPF-Phpy in different solvent also showed the solvation effect. The HOMO and LUMO calculation exhibited a slight delocalization, which leads to the solvation effect. The dipole moment calculation also proved that ODPF-Phpy has a large dipole moment. By contrast, ODPF-(Phpy)_2_ has a local orbital overlapping of HOMO and LUMO, and a small dipole moment. The PL spectra of ODPF-(Phpy)_2_ showed no obvious solvation effect.

**Figure S19.** TA data of M1 blending films. 2-D mapping of M1 blending films with (a) *n=*0, (b) *n=*0.2, (c) *n=*1 and (d) *n=*2. TA spectra with different decay time of M1 blending films with (e) *n=*0, (f) *n=*0.2, (g) *n=*1 and (h) *n=*2. The dynamics of different wavelength in M1 blending films with (i) *n=*0, (j) *n=*0.2, (k) *n=*1 and (l) *n=*2.

**Figure S20.** TA data of M2 blending films. 2-D mapping of M2 blending films with (a) *n=*0, (b) *n=*0.2, (c) *n=*1 and (d) *n=*2. TA spectra with different decay time of M2 blending films with (e) *n=*0, (f) *n=*0.2, (g) *n=*1 and (h) *n=*2. The dynamics of different wavelength in M2 blending films with (i) *n=*0, (j) *n=*0.2, (k) *n=*1 and (l) *n=*2.

**Table S4.** PLQY and conductivity of blending films.

| *n* | PLQY*^a^* (%) | | *σ^d^* (S cm^-1^) | |
| --- | --- | --- | --- | --- |
|  | M1 | M2 | M1 | M2 |
| 0  0.02  0.05  0.2  0.5  0.7  1  1.2  1.5  2 | 29.47*^b^*  67.78*^b^*  73.16*^b^*  78.34*^b^*  83.56*^b^*  91.04*^b^*  86.50*^c^*  71.98*^c^*  51.36*^c^*  52.39*^c^* | 32.67*^b^*  63.46*^b^*  72.14*^b^*  77.34*^b^*  82.41*^b^*  78.90*^b^*  74.86*^c^*  72.22*^c^*  71.71*^c^*  68.32*^c^* | 1.64×10^-9^  4.21×10^-9^  9.29×10^-9^  2.25×10^-8^  2.95×10^-8^  4.52×10^-8^  6.87×10^-8^  1.46×10^-7^  1.21×10^-7^  9.21×10^-8^ | 1.50×10^-9^  6.85×10^-9^  6.94×10^-9^  1.25×10^-8^  2.40×10^-8^  3.82×10^-8^  7.63×10^-8^  1.37×10^-7^  2.23×10^-7^  2.71×10^-7^ |

*^a^* Measured by TCSPC. *^b^* Ex=340 nm. *^c^* Ex=375 nm. *^d^* Device structure: ITO/Blending Film/Al. The conductivity was calculated by $\sigma=\frac{Id}{VA}$ , where *I* is the current, *V* is the voltage, *A* is the device area and *d* is the film thickness.

**Section S5. The stability of coordination compounds.**

**Figure S21.** The variable temperature PL spectra of (a) M1 with *n*=1 and (b) M2 with *n*=1. The normalized variable temperature PL spectra of (c) M1 with *n*=1 and (d) M2 with *n*=1.

In order to obtain the stability data of coordinated compound, we observed the changes of the components in the blending films indirectly by variable temperature PL spectra. The variable temperature PL spectra showed that the PL intensity of blending films would be decreased via increasing temperature. The PL spectrum of blending film started to change at 400 K. The emission peak started to blue shift and another peak at about 450 nm appeared. This phenomenon revealed the dissociation of the coordinated compound, leading to the decrease of film polarity and blue shift of the emission peak. From the TGA data of BCF (**Figure S6e**), we can observe that BCF started to decompose at about 400 K and the decomposition temperature (*T*_d_) of BCF was 135^o^C (408 K). In other words, it is the decomposition of BCF that cause the dissociation of the coordinated compound and the broken of N-B coordination.
